# Therapy-induced stress response is associated with downregulation of pre-mRNA splicing in cancer cells

**DOI:** 10.1186/s13073-018-0557-y

**Published:** 2018-06-27

**Authors:** Ksenia S. Anufrieva, Victoria О. Shender, Georgij P. Arapidi, Marat S. Pavlyukov, Michail I. Shakhparonov, Polina V. Shnaider, Ivan O. Butenko, Maria A. Lagarkova, Vadim M. Govorun

**Affiliations:** 1 Laboratory of Proteomics, Shemyakin-Ovchinnikov Institute of Bioorganic Chemistry of the Russian Academy of Sciences, Moscow, 117997 Russia; 2 Laboratory of Cell Biology, Federal Research and Clinical Center of Physical-Chemical Medicine of Federal Medical Biological Agency, Moscow, 119435 Russia; 3 Systems Biology Lab, Moscow Institute of Physics and Technology (State University), Moscow, Region, 141701 Russia; 4 Laboratory of Membrane Bioenergetics, Shemyakin-Ovchinnikov Institute of Bioorganic Chemistry of the Russian Academy of Sciences, Moscow, 117997 Russia; 5 Laboratory of Proteomic Analysis, Federal Research and Clinical Center of Physical-Chemical Medicine of Federal Medical Biological Agency, Moscow, 119435 Russia

**Keywords:** Alternative splicing, Cell cycle, Chemotherapy, DNA damage response, Spliceosome, Pladienolide B

## Abstract

**Background:**

Abnormal pre-mRNA splicing regulation is common in cancer, but the effects of chemotherapy on this process remain unclear.

**Methods:**

To evaluate the effect of chemotherapy on slicing regulation, we performed meta-analyses of previously published transcriptomic, proteomic, phosphoproteomic, and secretome datasets. Our findings were verified by LC-MS/MS, western blotting, immunofluorescence, and FACS analyses of multiple cancer cell lines treated with cisplatin and pladienolide B.

**Results:**

Our results revealed that different types of chemotherapy lead to similar changes in alternative splicing by inducing intron retention in multiple genes. To determine the mechanism underlying this effect, we analyzed gene expression in 101 cell lines affected by ɣ-irradiation, hypoxia, and 10 various chemotherapeutic drugs. Strikingly, оnly genes involved in the cell cycle and pre-mRNA splicing regulation were changed in a similar manner in all 335 tested samples regardless of stress stimuli. We revealed significant downregulation of gene expression levels in these two pathways, which could be explained by the observed decrease in splicing efficiency and global intron retention. We showed that the levels of active spliceosomal proteins might be further post-translationally decreased by phosphorylation and export into the extracellular space. To further explore these bioinformatics findings, we performed proteomic analysis of cisplatin-treated ovarian cancer cells. Finally, we demonstrated that the splicing inhibitor pladienolide B impairs the cellular response to DNA damage and significantly increases the sensitivity of cancer cells to chemotherapy.

**Conclusions:**

Decreased splicing efficiency and global intron retention is a novel stress response mechanism that may promote survival of malignant cells following therapy. We found that this mechanism can be inhibited by pladienolide B, which significantly increases the sensitivity of cancer cells to cisplatin which makes it a good candidate drug for improving the efficiency of cancer therapy.

**Electronic supplementary material:**

The online version of this article (10.1186/s13073-018-0557-y) contains supplementary material, which is available to authorized users.

## Background

Chemo- and radiotherapy combined with surgical removal of a tumor remain the most common treatment for cancer. However, this treatment is complicated by the development of drug resistance in cancer cells which leads to tumor recurrence [1, 2]. Therapy resistance can be attributed to a variety of mechanisms, such as DNA mutations, alterations in gene expression level, and abnormalities in pre-mRNA splicing [1–3]. The first two of these mechanisms are well studied, whereas the role of the pre-mRNA splicing regulation in development of chemotherapy resistance remains unclear [4].

Alternative pre-mRNA splicing is a process that affects more than 90% of multi-exon human genes. It allows cells to rapidly switch gene expression towards the production of various protein isoforms or noncoding transcripts and totally change the entire exome, proteome, and ultimately the cell phenotype [5–7]. Multiple pre-mRNA splicing abnormalities have been found in malignant cells leading to functional and nonfunctional changes in the proteome [8–11]. Further splicing perturbations were observed following chemotherapy. Several studies have described alternative splicing events that occur in cancer cells after treatment; however, most of these studies were focused on the function of specific genes rather than global changes in the transcriptome [12–17].

Most types of anticancer therapy frequently used in clinical practice are based on direct or indirect induction of DNA damage in target cells. The cellular response to DNA damage is a very complex process that includes stalling of RNA polymerase II, activation of multiple kinases (i.e., ATM, ATR, and DNA-PK), phosphorylation of histone H2AX, and cell cycle arrest [1, 18, 19]. These mechanisms allow tumor cells to repair DNA damage and survive following treatment. It has been recently reported that the splicing regulatory proteins may also play an important role in the DNA damage response and promote resistance to genotoxic stress by combining both splicing-dependent and splicing-independent mechanisms [20, 21].

Several small molecule splicing inhibitors were proposed lately to treat different types of cancer such as chronic lymphocytic leukemia, breast adenocarcinoma, prostate cancer, ovarian adenocarcinoma, and colon adenocarcinoma [22–24]. The most commonly used splicing inhibitors are pladienolide B and spliceostatin A. Both compounds bind to SF3B1 complex, blocking the spliceosome [25]. These small molecules were confirmed to effectively eliminate cancer cells both in vitro and in vivo; however, phase I clinical trials revealed multiple side effects due to high toxicity of the compounds [24].

In this study, we analyzed transcriptome data from patient-derived xenograft tumors and cancer cell lines to detect global changes in pre-mRNA splicing induced by multiple anticancer drugs. In addition, we comprehensively explored alterations in spliceosomal proteins. Particular, we used (I) mRNA microarray gene expression data from 101 cell lines derived from a variety of cancers, (II) quantitative phosphoproteome data, and (III) proteomic datasets of cells affected by various stress stimuli. We also tested the effect of the small molecule splicing inhibitor pladienolide B on the sensitivity of cancer cells to cisplatin.

## Methods

### Alternative splicing analysis

Unprocessed RNA-Seq reads were downloaded from the NCBI Gene Expression Omnibus (GEO) data repository (accession numbers GSE69405 and GSE89127; Table 1) [26]. To increase read quality before mapping, paired reads were trimmed using Trimmomatic (v. 0.35). The rMATS splicing tool [27] requires reads with a fixed length, so we trimmed reads from datasets GSE69405 and GSE89127 to the lengths of 96 and 90, respectively. We mapped trimmed RNA-Seq reads to hg19 of Gencode using STAR (v. 2.5.2b) according to recommendations from the STAR manual 2.4.0.1 with the following parameters: the maximum number of mismatches for paired reads was 4% of the read length, the maximum number of multiple alignments allowed for a read was 10, non-canonical junctions were removed, the minimum number of allowed splice overhangs was 8 for unannotated junctions and 1 for annotated junctions, the minimum intron length was 20, the maximum intron length was 1,000,000, and the number of “spurious” junctions was reduced. To compare splicing events before and after chemotherapy, we ran rMATS [27] splicing tool using the following parameters recommended by the developers: -c 0.0001 and -novel SS1 (multi-align reads are ignored as a default). Minor splicing differences were filtered out by thresholds of FDR < 0.05 and IncLevelDifference value > 5%. Splicing events were considered identical if they had the same coordinates and IncLevelDifference values had the same sign.

**Table 1:** The mRNA microarray gene expression and RNA-Seq data used in this study. The dataset title is used hereafter as a dataset identifier

Dataset	Cancer type	Time after treatment (h)	Number of samples	
			Control	After treatment
I. Platinum-based agents				
E-GEOD-38122 [103]	Hepatocyte carcinoma	24	3	3
GSE47980 [104]	Melanoma	24	9	9
GSE38545	Ovarian cancer	24	3	3
GSE13525 [105]	Ovarian cancer	24	2	2
E-GEOD-8057 [106]	Ovarian cancer	24	4	4
GSE51952 [107]	Hepatocyte carcinoma	24	3	3
GSE66493	Glioblastoma	24	3	3
GDS3910 [108]	Breast cancer	11	2	2
E-MTAB-3645 [109]	Ovarian cancer	72	3	3
II. Paclitaxel				
E-GEOD-50831 [110]	Ovarian cancer	24	63	63
E-GEOD-50830 [110]	Endometrial adenocarcinoma	24	55	57
E-GEOD-50811 [110]	Breast cancer	24	79	81
III. Irradiation (two meta-analyses for cancer and embryonic cells)				
E-GEOD-59732 [111]	Breast cancer	24	48	48
E-GEOD-59861 [112]	Skin fibroblasts	0, 3, 6, 12, 24	4	12
IV. Hypoxia				
E-GEOD-18494 [113]	Hepatocyte carcinoma, glioblastoma, breast cancer	12	9	9
E-GEOD-53012 [114]	Prostate cancer, ovarian cancer, melanoma	72	9	9
E-MTAB-3645 [109]	Ovarian cancer	72	3	3
E-GEOD-17188 [115]	Breast cancer	24	4	4
V. Tyrosine kinase inhibitors				
E-TABM-585 [116]	Lung cancer	20	21	27
VI. Topoisomerase inhibitors				
E-GEOD-47013 [117]	Multiple myeloma	–	3	3
E-GEOD-13477 [118]	Breast cancer	24	2	2
E-GEOD-19638 [119]	Breast cancer	–	2	2
E-GEOD-39870 [120]	Breast cancer	–	3	3
Splicing analysis				
GSE69405 [37]	Lung adenocarcinoma	48	12	12
GSE89127 [34]	Gastric carcinoma, lung adenocarcinoma, melanoma, urinary bladder carcinoma	48	18	18
Spliceostatin				
GSE72156 [58]	Cervical cancer	–	3	3
Pladienolide				
E-GEOD-67770 [57]	Ovarian cancer	–	2	2

### Gene expression analysis

In total, 26 microarray datasets (for dataset accession numbers see Table 1 and Additional file 1) were loaded from two repositories: NCBI GEO [26] and EMBL-EBI ArrayExpress [28]. Raw microarray data from Affymetrix microarrays (CEL files) were processed using the affy R/Bioconductor package [29] with the following parameters: normalization—quantiles, background correction—rma, probe specific correction—pmonly, and summary method—medianpolish. The final data were already log-transformed. The lumi [30] and limma [31] R/Bioconductor packages were used to process the data obtained from Illumina Beadchips. The parameters were as follows: variance stabilization—log2 and normalization—quantiles. Before that, an initial detection *p* value threshold of 0.05 was chosen based on Illumina recommendations. rMeanSignal and gMeanSignal were taken as the values of the Agilent microarray signal. For further analysis of the data obtained from Agilent microarrays, the logarithm of the rMeanSignal-to-gMeanSignal ratio was normalized using the quantile normalization method.

Gene expression was compared between two group of cells (intact cells and exposed to stress: chemotherapy, radiotherapy, or hypoxia) using the limma R/Bioconductor package [31].

### Gene expression results aggregation

The advantage of our analysis was that we combined *p* values of separate datasets with the use of Wilkinson’s method [32] for meta-analysis of significance values from metap R/Bioconductor package [33]. First, we considered *p* values of differentially expressed genes using the limma R/Bioconductor package [31], and then, we combined *p* values with the method of Wilkinson and made a correction for multiple testing with the FDR method. Methods for combining *p* values gives a statistician flexibility, since they require minimal information and assumptions from gene expression studies, and thus, we did not account for the variability in the datasets or microarray platforms.

Meta-analysis of cisplatin, hypoxia, and topoisomerase inhibitor actions (Table 1, Additional file 1) were processed according to the above scheme of meta-analysis with a *p* value cutoff threshold of 0.05. We picked only those genes that were exclusively up or downregulated in all the studies compared to their corresponding controls. Gene expression levels were combined between studies on different platforms using RefSeq ID obtained from the microarray annotation file. To analyze the effect of paclitaxel (Table 1, Additional file 1), all corresponding samples were combined into one set as these data came from the same source using Affymetrix GeneChip Human Genome U133 Plus 2.0 software. Differentially expressed genes were identified without meta-analysis techniques using only the limma R/Bioconductor package [31]. We could not find a sufficient number of datasets for the effect of radiation and tyrosine kinase inhibitors’ effect in public repositories, and therefore, differentially expressed genes were independently identified for each separate dataset using only the limma R/Bioconductor package [31].

### Сell cultures

Human ovarian (SKOV3), breast (MCF7), colorectal (HT29), cervix (Hela) adenocarcinoma, lung carcinoma (A549), hepatocellular carcinoma (HepG2), and glioblastoma (U87MG) cell lines were grown in DMEM (Sigma) supplemented with a 1% penicillin/streptomycin mixture (Gibco), 2 mM glutamine (Gibco), and 10% fetal bovine serum (HyClone) in a humidified 5% CO2 incubator at 37 °C. The cells were checked for signs of mycoplasma contamination.

### LC-MS/MS

The LC-MS/MS analysis of SKOV3 cell lysates was performed in three replicates using a TripleTOF 5600+ mass spectrometer with a NanoSpray III ion source (ABSciex) coupled to a NanoLC Ultra 2D+ nano-HPLC system (Eksigent). The HPLC system was configured in trap-elute mode. For sample loading buffer and buffer A, we used a mixture of 98.9% water, 1% methanol, and 0.1% formic acid (*v*/*v*). Buffer B was 99.9% acetonitrile and 0.1% formic acid (*v*/*v*). Samples were loaded on a Chrom XP C18 trap column (3 μm, 120 Å, 350 μm × 0.5 mm; Eksigent) at a flow rate of 3 μl/min for 10 min and eluted through a 3C18-CL-120 separation column (3 μm, 120 Å, 75 μm × 150 mm; Eksigent) at a flow rate of 300 nl/min. The gradient was increased from 5 to 40% buffer B over 90 min followed by 10 min at 95% buffer B and 20 min of reequilibration with 5% buffer B. Between different samples, two blank 45-min runs consisting of 5 to 8 min waves (5% B, 95%, 95%, 5%) were required to wash the system and prevent carryover.

The information-dependent mass spectrometry experiments included one survey MS1 scan followed by 50 dependent MS2 scans. The following MS1 acquisition parameters were used: the mass range for MS2 analysis was 300–1250 m/z, and the signal accumulation time was 250 ms. The ions used for the MS2 analysis were selected based on intensity with a threshold of 200 cps and a charge state from 2 to 5. The following MS2 acquisition parameters were used: the resolution of the quadrupole was set to UNIT (0.7 Da), the measurement mass range was 200–1800 m/z, and the signal accumulation time was 50 ms for each parent ion. Collision-activated dissociation was performed using nitrogen gas and by ramping collision energy from 25 to 55 V within a signal accumulation time of 50 ms. Analyzed parent ions were sent to a dynamic exclusion list for 15 s to obtain MS2 spectra at the chromatographic peak apex. A β-galactosidase tryptic solution (20 fmol) was run with a 15-min gradient (5–25% buffer B) between samples to calibrate the mass spectrometer and control overall system performance, stability and reproducibility.

Descriptions of further bioinformatics (pathway analysis, time clusterization analysis, co-regulation analysis, LC-MS/MS protein identification) and experimental methods (SDS-PAGE, in-gel trypsin digestion, cell proliferation assay, flow cytometry, immunofluorescence analysis and western blotting) are provided in Additional file 2.

## Results

### The action of different chemotherapeutic drugs leads to intron retention in genes involved in splicing regulation

**Fig. 1:**
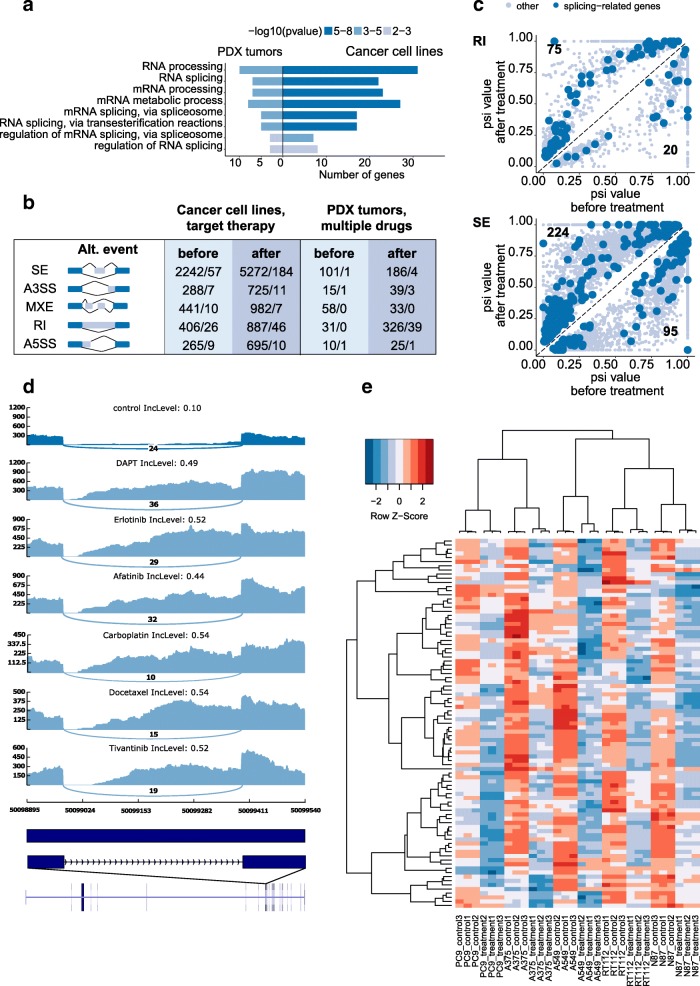
Analysis of pre-mRNA splicing changes in cancer cell lines and xenografts after chemotherapy. **a** Enrichment analysis of genes with common differential splicing events induced by therapy. Left side: lung adenocarcinoma patient-derived xenografts (PDX) treated with carboplatin, docetaxel, afatinib, BEZ235, BKM120, DAPT, erlotinib, tivantinib, and selumetinib; dataset GSE69405. Right side: A375, A549, H3122, N87, PC9, RT112 cell lines treated with erlotinib, crizotinib, trametinib, lapatinib, vemurafenib, BGJ398; dataset GSE89127. The STRING database was used for Gene Ontology Biological Processes analysis. *p* value is indicated with a color scale. **b** Summary of alternative splicing events observed in GSE89127 and GSE69405 datasets (before slash: total number of splicing events; after slash: common splicing events appeared in at least half of the samples). Events: SE—skipped exon, A5SS—alternative 5′ splice site, A3SS—alternative 3′ splice site, RI—retained intron, MXE—mutually exclusive exons. **c** Scatter plot representing the intron retention (upper panel) and exon skipping (lower panel) events detected in the GSE89127 dataset before and after chemotherapy. Splicing events in spliceosomal genes are illustrated with a dark-blue color. **d** Sashimi plots for the splicing factor RBM6 in untreated cancer cells (dark blue) and in cancer cells that were treated with different chemotherapeutic drugs (light blue). The inclusion level (IncLevel) indicates the splicing status of the intron. **e** Heat map demonstrating the changes in the expression of spliceosomal genes (*Z*-score) after chemotherapy. Clusterization of expression data was made before scaling data (*Z*-score transformation)

To determine how pre-mRNA splicing in cancer cells is affected by chemotherapy, we analyzed publicly available transcriptome datasets (GSE89127) that represent melanoma, lung cancer, gastric carcinoma, and bladder carcinoma cell lines (A375, A549, H3122, N87, PC9, RT112) treated with multiple kinase inhibitors (erlotinib, crizotinib, trametinib, lapatinib, vemurafenib, BGJ398) [34] (Table 1). In total, we detected 12,203 altered alternative splicing events in intact cells and cells exposed to kinase inhibitors. Interestingly, among them, 367 splicing events were observed in at least half of the cell lines after the therapy (Additional file 3A). Analysis of genes affected by alternative splicing revealed significant number of genes from the pre-mRNA splicing pathway (Fig. 1a). Thus, we assumed a regulatory feedback mechanism between the spliceosomal proteins and their targets. In other words, therapy-induced alterations in pre-mRNA splicing affect spliceosomal genes, which in turn may provoke further splicing perturbations. To assess possible effects of splicing changes on gene expression, we investigated types of alternative splicing events that occurred after therapy. Our analysis revealed two common splicing alterations: exon skipping (184 events) and intron retention (46 events) (Fig. 1b). In contrast to exon skipping, intron retention was observed more frequently after the therapy, i.e., in most cases the intron inclusion level was higher in treated samples than in untreated control cells (Fig. 1c). Analysis of transcripts with retained introns demonstrated that all of them contain multiple in frame stop codons (Additional file 3B). Therefore, they could not be translated into functional proteins [35, 36]. Our data indicate that the spliceosome pathway might be suppressed by chemotherapy.

To further examine our hypothesis in a more clinically relevant model, we analyzed a publicly available transcriptome dataset (GSE69405) that represents 12 lung adenocarcinoma patient-derived xenograft (PDX) tumors prior to and after treatment with chemotherapeutic drugs, including carboplatin, docetaxel, afatinib, BEZ235, BKM120, DAPT, erlotinib, tivantinib, and selumetinib (Table 1). These data were used in the original study to compare gene expression levels without paying attention to mRNA splice variants [37]. Strikingly, we found that different drugs induced similar alterations in pre-mRNA splicing. This trend was further confirmed by PCA clustering of inclusion level differences between treated and untreated samples (Additional file 4: Figure S1). We detected 824 altered alternative splicing events in treated cells (Fig. 1b), and, similar to the previous dataset, the most frequent type of alternative splicing variants was intron retention. In total, more than half of samples had 47 common splicing events and 39 of them involved in intron retention (Fig. 1b, Additional file 3C, D).

As in the experiments conducted on cancer cell lines, in patient-derived tissues, a chemotherapy induces splicing alterations in genes involved in the spliceosome pathways (Fig. 1a). We observed intron retention in several essential splicing factors, including RBM6, HNRNPA2B1, RBM39, RBM5, SRRM1, SRSF5, and SRSF7 (Fig. 1d, Additional file 3E). Interestingly, treatment of cancer cells with different chemotherapeutic drugs resulted in retention of the same introns in each of these genes.

### Chemotherapy and irradiation lead to a concerted change in the expression of spliceosomal and cell cycle genes

**Fig. 2:**
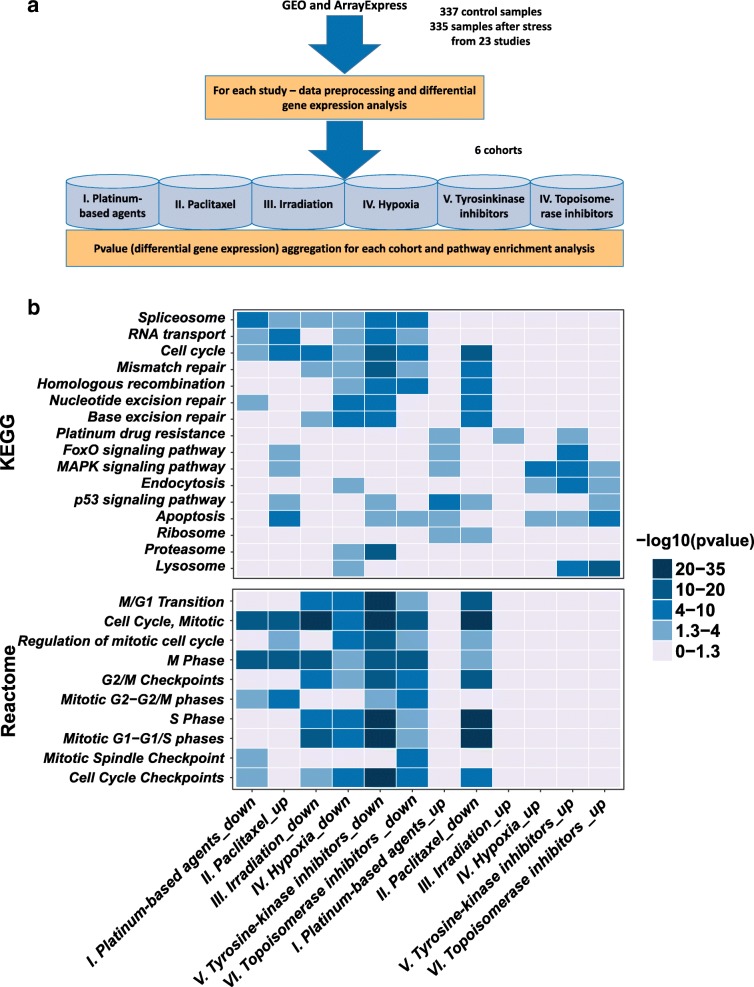
Changes in gene expression in cancer cells in response to different stress conditions. **a** Workflow of the meta-analysis of mRNA microarray gene expression data. **b** Heat maps of selected pathways obtained by KEGG (top panel) and Reactome (bottom panel) enrichment analyses of up and downregulated genes in each of the six meta-analyses of gene expression

Having discovered that significant changes in pre-mRNA splicing in response to the action of various types of chemotherapeutic drugs, we next examined global changes in gene expression. We decided to perform analysis of mRNA levels in the same dataset that was used previously for evaluation of alternative splicing differences (GSE89127). It has revealed a significant downregulation of splicing-related gene levels in all cancer cell lines after chemotherapy (Fig. 1e). To expand this result to larger number of samples and multiple types of cancers, we performed six meta-analyses of mRNA microarray gene expression profiles of different cell lines exposed to various stress stimuli, including DNA-damaging agents (i.e., platinum-based drugs, gamma irradiation, and topoisomerase inhibitors), tyrosine kinase inhibitors, and taxanes (Fig. 2a). A detailed description of these datasets is presented in Table 1 and Additional file 1.

Our meta-analysis (Fig. 2b (top panel) and Additional file 5) revealed multiple downregulated and upregulated pathways, which were exclusively observed for certain drugs. For example, platinum-based compounds promoted the expression of genes responsible for apoptosis and those involved in the platinum drug resistance pathway (Additional file 5). While topoisomerase and tyrosine kinase inhibitors provoked the expression of apoptotic, lysosomal, and MAPK signaling genes. The only two pathways that were commonly affected in all tested datasets regardless of the treatment used were the cell cycle regulation (especially M phase regulation) and the pre-mRNA splicing pathways. The level of gene expression in these two pathways was significantly downregulated after treatment with anticancer drugs.

In clinical practice, platinum-based drugs are often combined with taxanes [38–40]. The cytostatic effect of taxanes is mainly associated with enhanced microtubule formation and stabilization [41]. The stimulation of microtubule formation temporarily activates CDK1 and triggers the G2/M transition, which is followed by mitotic arrest [42]. It is in line with our meta-analysis of paclitaxel-treated cancer cells. The levels of genes involved in M phase were significantly upregulated, while those associated with the S phase and the G1/S transition were downregulated (Fig. 2b (bottom panel), Additional file 5). These data are consistent with previous findings [41, 43]. Interestingly, we also observed a substantial increase in the transcription levels of genes involved in spliceosome assembly and regulation and in RNA transport.

Next, we studied the response of cancer cells to hypoxia, which is one of the natural stress factors for cancer cells. The result of corresponding dataset analysis was not so surprising: the spliceosomal and cell cycle progression genes were found in the cluster of downregulated pathways (Fig. 2b (top panel), Additional file 5). In particular, the level of transcripts associated with M phase of the cell cycle were significantly downregulated (Fig. 2b (bottom panel)).

Taken together, in our analysis of gene expression in 101 cell lines that were affected by six different types of stress, we identified only two pathways that were altered in all tested samples. The downregulation of cell cycle-related pathways was expected since it has recently been shown that stress induces retardation or arrest of specific cell cycle phases [43, 44]. Surprisingly, in addition to cell cycle changes, we consistently observed concerted changes in the expression of spliceosomal genes. To the best of our knowledge, this phenomenon has not been reported previously.

### Clusterization of time-series gene expression data revealed the same dynamics in the regulation of genes involved in cell cycle and splicing

**Fig. 3:**
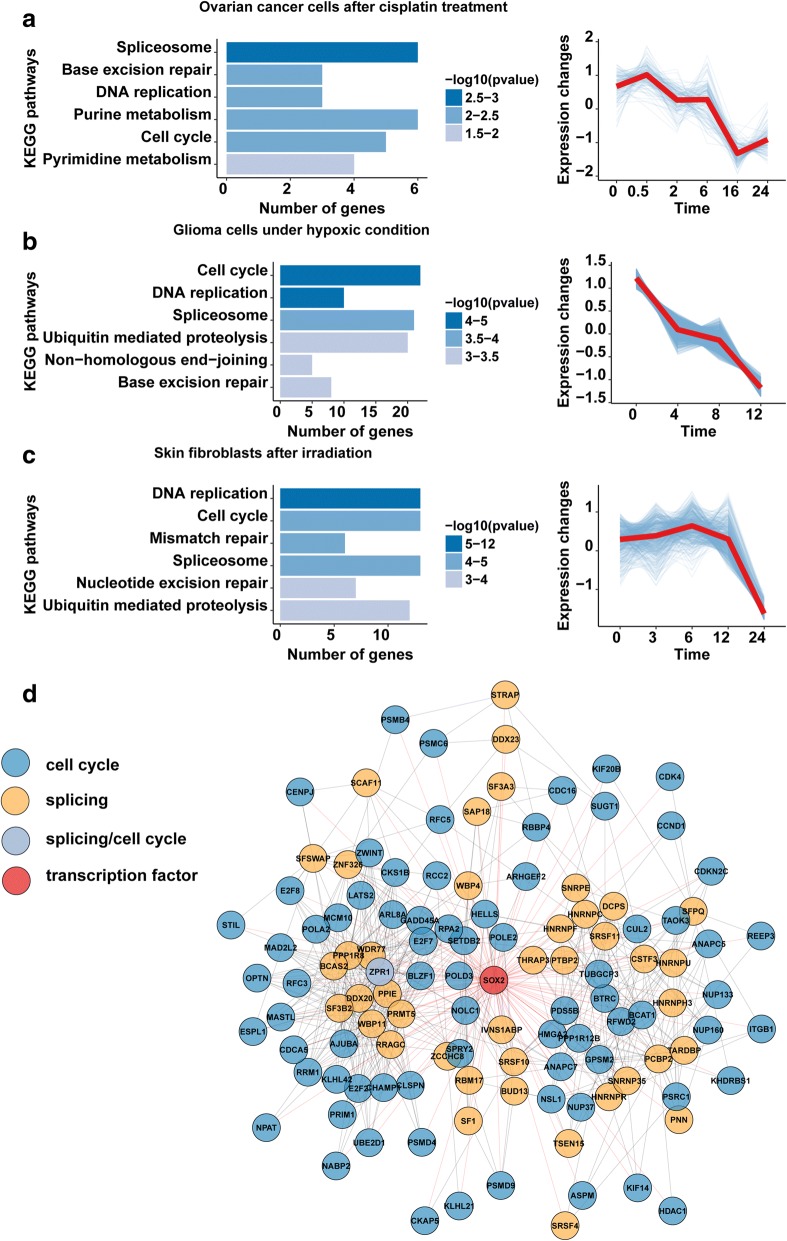
Different types of clusterization show concerted changes in the expression of spliceosomal and cell cycle genes. **a–с** Time clusterization of gene expression data (right panel) and subsequent pathway enrichment analysis (left panel) of clusters with highly represented spliceosomal genes. The clusters were constructed based on the following datasets: **a** E-GEOD-8057, **b** E-GEOD-18494, and **c** E-GEOD-59861. Blue lines represent *z*-score values of gene expression in each cluster. The red line is a mean value of *z*-score values of a cluster. **d** Graph representing the common transcription factor SOX2 that may induce concerted changes in the expression of pairs of mitotic and splicing genes after a course of chemotherapy. Solid black lines connect a pair of co-expressed genes, and red lines connect transcription factors with their target genes. Additional file 4: Figure S2*A, B* shows similar graphs for the transcription factors GFI1B and TARDBP

To understand the dynamics of changes in the expression of spliceosomal genes following exposure to different stress factors, we implemented soft clustering (i.e., fuzzy c-means clustering). We analyzed two datasets containing at least four time points after the beginning of the treatment: an ovarian cancer cell line treated with cisplatin (E-GEOD-8057) and a glioma cell line subjected to hypoxia (E-GEOD-18494). For both datasets, the cluster analysis clearly showed that spliceosomal gene levels were gradually downregulated (Fig. 3 a, b; Additional file 6). In spliceosomal gene clusters, we also found genes that were associated with the cell cycle and DNA replication pathways (Additional file 6). In particular, cell cycle genes involved in M and S phase transitions were significantly downregulated. The simultaneous decrease in the expression levels of cell cycle- and spliceosome-related genes indicates that the downregulation of splicing is not a consequence of cell cycle arrest. It is more likely represents an independent mechanism of the cell response to stress.

Next, we were interested whether the concerted downregulation of spliceosomal and cell cycle genes is unique to cancer cells. Therefore, we conducted the soft clustering analysis of the transcriptome of gamma-irradiated normal skin fibroblasts (dataset E-GEOD-59861) (Fig. 3c, Additional file 6). Strikingly, results showed that spliceosomal genes were significantly downregulated in gamma-irradiated fibroblasts and the clusters enriched with spliceosomal genes also contained genes involved in DNA replication and M and S phase cell cycle transitions. Our results imply that observed concerted downregulation of expression levels of genes involved in splicing and cell cycle regulation is not specific for cancer cells.

### Co-regulation network clusterization revealed common transcription factors for genes involved in mitosis and splicing

Since spliceosomal genes and genes involved in the M phase of the cell cycle were changed in a concerted manner, we wondered whether these genes were regulated by the same transcription factors. To answer this question, we performed a co-expression analysis of genes differentially expressed in cancer cells exposed to chemotherapeutic drugs and created a co-regulatory network based on ChIP-seq from the ReMap database. We selected four datasets (E-GEOD-66493, GSE13525, GSE66493, and GSE47856) that describe the effects of platinum agents on gene expression in different cancer cell lines.

For each dataset, we independently identified differentially expressed splicing- and mitotic-related genes and chose pairs with Spearman correlation coefficients greater than 0.7 and FDR-corrected *p* value < 0.05. Next, we determined transcription factors that bind to the promoter regions of the co-expressed genes in chosen pairs (for more detail see Additional file 2). Our analysis revealed three transcription factors that were common for all datasets, namely, SOX2, GFI1B, and TARDBP (Fig. 3d, Additional file 4: Figure S2A, B). We assumed that these factors might be responsible for concerted gene expression changes in spicing- and mitotic-related genes.

### Chemotherapy induces phosphorylation and secretion of spliceosomal proteins

**Fig. 4:**
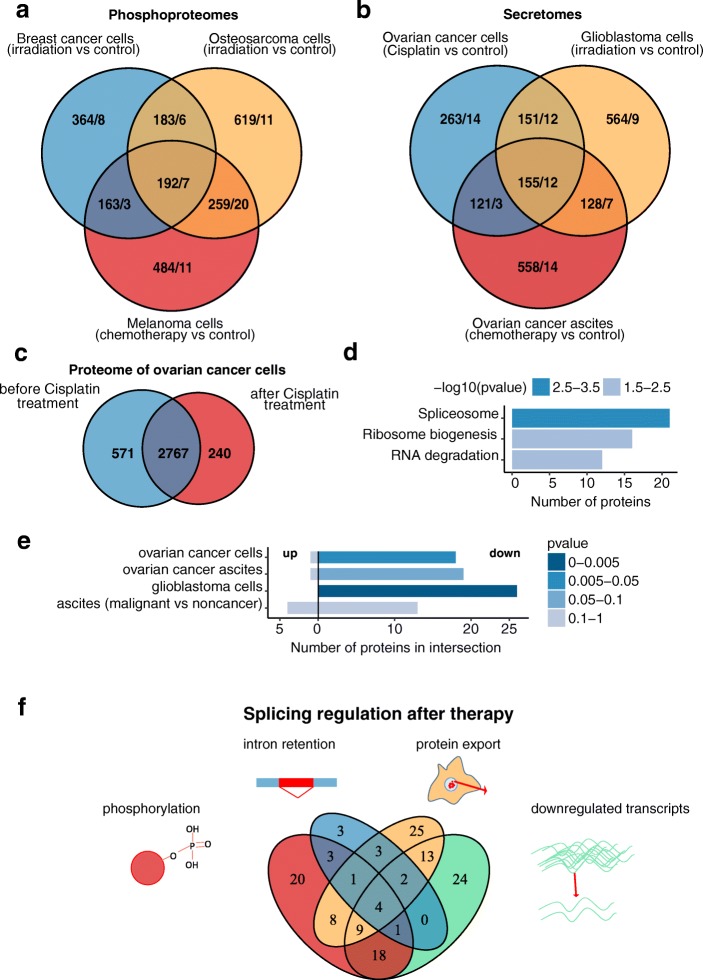
Changes in proteomic profiles induced by different stress conditions. **a** Comparison of proteins that were phosphorylated in a breast cancer cell line after gamma-irradiation [49] (blue); in an osteosarcoma cancer cell line after gamma-irradiation [48] (orange) and in a melanoma cancer cell line after neocarzinostatin [50] (red). The number of spliceosomal proteins in a given sector is shown after the slash. **b** Venn diagram of upregulated proteins in therapy-induced secretomes of ovarian cancer cells (blue circle), glioblastoma cells (orange circle), and ovarian cancer ascites obtained from patients after the course of chemotherapy (red circle) [51, 52]. The number of spliceosomal proteins in a given sector is shown after the slash. **c** Venn diagram representing the proteins identified in SKOV3 cells before (blue) and after (red) cisplatin treatment. **d** Results of the enrichment analysis of proteins (from “C”) for which abundance was decreased by more than twofold after chemotherapy. **e** Results of Fisher’s test of the intersection between differentially secreted proteins (derived from data we reported in [51, 52]) and the hits from siRNA screening (based on the study by Paulsen et al. [53]). **f** Intersection of the lists of the spliceosomal genes with a decrease in expression (green circle, according to our meta-analyses of microarray data), intron retention in transcripts (blue circle, according to our meta-analyses of RNA-Seq data), upregulated secretion of the corresponding proteins (orange circle, according to our previous proteomic data) and upregulated protein phosphorylation (red circle, according to the analysis of phosphoproteomics data) observed after treatment with chemotherapeutic drugs

In addition to the transcriptional control of gene expression, the activity of spliceosomal proteins can also be regulated at the proteome level by post-translational modifications. It has been shown that splicing can be inhibited by the phosphorylation of spliceosomal proteins as negatively charged phosphate groups obstruct binding between proteins and negatively charged RNA molecules [45–47]. Therefore, we analyzed three publicly available phosphoproteomes of cancer cells that were subjected to DNA damage (gamma irradiation or neocarzinostatin) [48–50] and compared proteins that were phosphorylated before and after treatment. According to our analysis, spliceosomal proteins were significantly phosphorylated after the therapy. In total, we identified 66 spliceosomal proteins with high post-treatment phosphorylation levels (Fig. 4a). These data favor the possibility that DNA damage may suppress the catalytic activity of spliceosomal proteins in cells.

In addition, several studies have shown that following exposure to chemo- [51] or radiotherapy [52] cancer cells specifically secrete spliceosomal proteins into the extracellular space. Consistent with these observations, we have previously identified multiple spliceosomal proteins in ovarian cancer ascites (extracellular fluids that serve as a natural medium for cancer cells) obtained from patients after treatment [51]. The proteins for which secretion increased more than twofold after radiotherapy of glioblastoma cells in vitro [52] and after chemotherapy of ovarian cancer cells both in vitro (cisplatin treatment) and in vivo (ascites from patients after combined course of chemotherapy) [51] are shown on Fig. 4b. In these datasets, we identified more than 30 spliceosomal proteins that could be exported from cancer cells in response to therapy. We validated these data by western blotting with antibodies against several spliceosomal proteins U2AF65, U2AF35, and RBM11 (Additional file 4: Figure S3).

To experimentally confirm the results of our meta-analyses, we performed LC-MS/MS-based proteome profiling of SKOV3 ovarian cancer cells before and 24 h after treatment with cisplatin. In total, 3578 proteins were identified in our experiment (Fig. 4c, Additional file 7). Proteins were considered differentially present if their abundance was changed by more than twofold. We found that the abundance of 366 proteins were increased and the abundance of 922 proteins were decreased after cisplatin treatment. Data analysis using the KEGG database revealed that only three pathways were significantly altered in the treated cells (Fig. 4d). Interestingly, the spliceosome-related pathway proteins were substantially downregulated in accordance with our results of gene expression analyses.

### Small molecule splicing inhibitor impairs DNA damage response

Based on the results on our transcriptomic and proteomic analyses, we conclude that alterations in pre-mRNA splicing are induced by multiple mechanisms during therapy: altered gene expression, pre-mRNA splicing dysregulation, protein post-translational modification and secretion. However, our analysis does not explain the biological reason for these changes. The role of the spliceosome in the DNA damage response has lately been demonstrated in several studies [53, 54]. Paulsen and colleagues performed full-genomic siRNA screening of human cells and identified that downregulation of genes involved in pre-mRNA splicing induced the highest level of H2AX phosphorylation [53], which serves as an early marker of the DNA damage response [55, 56]. We collated the results of siRNA screening for histone H2AX phosphorylation [53] with the results of our meta-analysis of gene expression and proteomic data [51, 52] (Fig. 4e). Fisher’s test showed that the proteins secreted by DNA-damaged cells are encoded by genes for which insufficient expression is perceived by the cell as a DNA repair signal (Fig. 4e). Therefore, the downregulation of several spliceosomal proteins that was observed during therapy may activate DNA reparation.

**Fig. 5:**
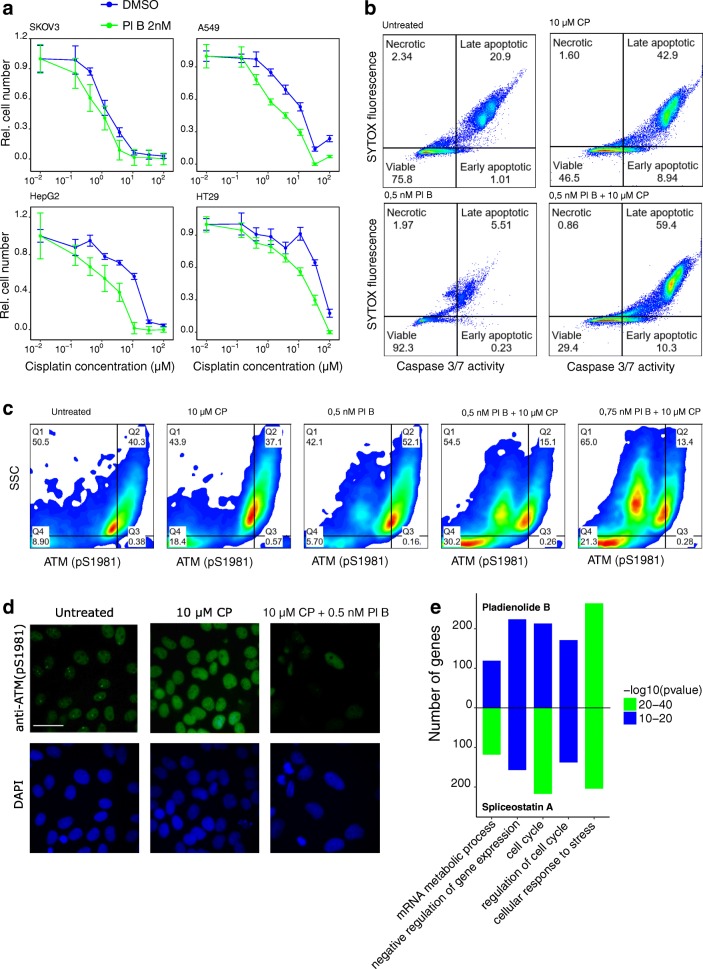
Pladienolide B impairs pre-mRNA splicing and increases the sensitivity of cancer cells to cisplatin. **a** Viability assay of SKOV3, A549, HepG2, and HT29 cells that were pretreated with 2 nM pladienolide B (2 days) following treatment with different concentrations of cisplatin (4 days). **b** FACS analysis of caspase 3/7 and SYTOX staining of A549 cells treated with 0.5 nM pladienolide B, 10 μM Cisplatin or both drugs together. **c** FACS analysis of phospho ATM staining of SKOV3 cells that were cultivated with different concentrations of pladienolide B (2 days) and subsequently treated with 10 μM Cisplatin (1 day). **d** Representative immunofluorescence images of SKOV3 cells stained for phosphoATM (S1981) (green) and with DAPI (blue) after treatment with 10 μM Cisplatin in the presence or absence of 0.5 nM Pladienolide B. Scale bar: 50 μm. **e** Enrichment analysis of genes affected by differential splicing events before and after treatment with splicing inhibitors: pladienolide B (upper part; E-GEOD-67770) and spliceostatin A (lower part; GSE72156). The STRING database was used for Gene Ontology Biological Processes analysis

Based on these data, we proposed that the changes in alternative splicing and downregulation of spliceosomal transcripts and proteins that were observed after therapy may help cancer cells survive after genotoxic stress. To test this hypothesis, we treated ovarian (SKOV3), breast (MCF7), colorectal (HT29), cervix (Hela) adenocarcinoma, lung carcinoma (A549), hepatocellular carcinoma (HepG2), and glioblastoma (U87MG) cell lines with sublethal doses of the small molecule splicing inhibitor pladienolide B for 2 days. After treatment, we evaluated the sensitivity of these cells to the DNA damaging drug cisplatin using an MTT assay (Fig. 5a, Additional file 4: Figure S4A). This experiment revealed that nanomolar concentrations of pladienolide B significantly increase the sensitivity of cancer cells to cisplatin (Fig. 5b, Additional file 4: Figure S4B).

To determine how splicing inhibitors affect cancer cells, we analyzed RNA sequencing data from ovarian adenocarcinoma cells (TOV21G; E-GEOD-67770) [57] treated with pladienolide B and cervix adenocarcinoma cells (Hela; GSE72156) [58] treated with another potent splicing inhibitor spliceostatin A with the same mechanism of action as pladienolide B. Figure 5e demonstrates that both inhibitors predominantly affect splicing of genes involved in the cellular stress response and cell cycle regulation. We confirmed these data through cell cycle analysis of HT29 and SKOV3 cells treated with pladienolide B (Additional file 4: Figure S4C).

Tressini and colleagues have recently demonstrated that spliceosomal complexes play an important role in the activation of ATM kinase and subsequent DNA repair [19]. This signaling pathway is activated by ATM autophosphorylation on Ser1981 [18]. Therefore, having obtained data indicating that pladienolide B impairs cell response to stress, we decided to investigate its effect on the level of phosphorylated ATM after DNA damage. FACS analysis and immunofluorescence microscopy demonstrated that pretreatment with pladienolide B dramatically decreases cisplatin-induced ATM phosphorylation in SKOV3, Hela, HT29, and A549 cells (Fig. 5c, d and Additional file 4: Figure S4D). Based on these data, we conclude that regulation of pre-mRNA splicing is important for ATM-dependent DNA damage response while dysregulation of spliceosomal machinery induced by a splicing inhibitor impairs ATM signaling and increases the sensitivity of cancer cells to genotoxic stress.

## Discussion

Dysregulation of pre-mRNA splicing can lead to the appearance of protein isoforms that contribute to the initiation and progression of tumors and cancer resistance to chemotherapy [5, 59, 60]. In addition to the imbalance in splicing regulation that is inherent to cancer cells (as opposed to normal cells) [35, 61–63], the effectiveness and precision of pre-mRNA splicing are further affected by chemotherapy [4, 64]. Recent global analysis of splicing changes in tumor samples in comparison with normal tissues from TCGA collection revealed that aberrant alternative splicing often affected the functional domains of proteins, which frequently mutated in cancer [7]. The origin of cancer-related splicing changes remains mostly unknown. On the one hand, the global tendency of splicing dysregulation in cancer has been demonstrated in several excellent studies that revealed various cancer-associated mutations in spliceosomal genes that impair pre-mRNA splicing [5, 65, 66]. However, even in the absence of mutations in splicing factors, more intron retention events occur in cancer tissues than in normal cells [35]. On the other hand, many splicing factors are frequently overexpressed in multiple cancers [67]. Intriguingly, a growing number of studies demonstrates the functions of spliceosomal proteins that are not directly related to the splicing process such as DNA repair [68], R-loop formation [69], telomere elongation [20], mRNA export from the nucleus [70], and M-phase regulation [71, 72].

Here, we comprehensively studied differences in alternative splicing and changes in the expression of spliceosomal genes in cancer cells following treatment with a variety of chemotherapeutic drugs. We analyzed publicly available poly(A)+ RNA-sequencing data and demonstrated that the most frequent alternative splicing event after chemotherapy was intron retention. Inefficient intron removal indicates less intense pre-mRNA splicing. It is important to note that poly(A)+ RNA-sequencing data could underestimate the extent of alternative splicing events because poly(A)+ RNA-seq depletes some fraction of intronic reads [73]; therefore, the real intron retention might be even higher then detected during our analysis. Our data strongly support the results of the meta-analyses, in which we observed a decrease in the expression of spliceosomal genes following exposure to several types of stress stimuli. To the best of our knowledge, this is the first study demonstrating an increase in the number of intron retention events in various cancer cells following different types of chemotherapy. Recently, one group of studies tried to recognize the regulation of one or a few transcripts following therapy [12–17]. Other studies focused only on exon inclusion/skipping events using splicing-sensitive microarrays. For example, it has been shown that camptothecin altered alternative splicing of genes involved in splicing regulation in MCF-7 and HCT116 cell lines [64, 74, 75]. Only a few works interrogated entire transcriptome changes after stress response. It has been reported that hypoxia and cisplatin treatment also affected the splicing of spliceosomal genes in breast cancer cell line [4, 76]. In our systematic analysis, we observed a similar enrichment pattern of alternative splicing changes across various cancer cell lines derived from different tumor types after exposure to various chemotherapeutic drugs. We showed that genes most affected by intron retention were genes that encodes spliceosomal proteins. Intron retention usually leads to RNA degradation and may therefore provide an additional mechanism by which downregulation of spliceosomal proteins is facilitated within the cell.

Intriguingly, the results of our meta-analyses of gene expression and the results of time clustering showed that spliceosomal genes and genes involved in the mitotic part of the cell cycle were simultaneously downregulated in different cell lines following exposure to various stresses. The only exception was the effect of taxanes. This group of drugs stimulates the G2/M transition and as a result upregulates genes involved in M phase. Importantly, we observed a simultaneous increase in spliceosomal genes after treatment with taxanes. Therefore, we observed concerted changes in the expression of spliceosomal and cell cycle-related genes following all types of treatment. An interplay between the cell cycle and the spliceosome has been demonstrated in many organisms, including yeast [77–82], fruit flies [83, 84], chickens [54], mice [85], and humans [54, 72, 86–90]. In human cells, inhibition of spliceosomal gene expression using siRNA led to multiple defects in cell cycle progression, most of which were related to mitosis [72, 86–88]. In addition, it has been shown that a lack of spliceosomal components causes cell cycle arrest in S and G2 phases [91, 92]. Furthermore, a connection between alternative splicing and cell cycle regulation has been demonstrated by Tsai and colleagues, who showed that alterations in pre-mRNA splicing are correlated with expression changes of genes involved in cell cycle regulation in cancer cells [93]. Therefore, there is a strong interplay between pre-mRNA splicing and proper cell cycle progression.

Our results show that the key transcription factors that regulate the concerted changes in the expression of spliceosomal and cell cycle genes were the proto-oncogenes SOX2 and GFI1B and the transcriptional repressor TARDBP. Changes in these transcription factors may play an oncogenic role in tumor formation [94–96]. SOX2 has been implicated in growth, tumorigenicity, drug resistance, and metastasis in at least 25 different cancers [97], TARDBP is involved in apoptosis and cell division, while GFI1B positively regulates c-Myc expression and increases the proliferation rate of cancer cells [98].

We showed that the amount of spliceosomal proteins in cells decreased after treatment with chemotherapeutic drugs through different molecular mechanisms. In addition to reduced expression and disturbances in splicing of spliceosomal genes, cancer cells secrete spliceosomal proteins into the extracellular space after a course of chemotherapy. Consistent with this observation, our LC-MS/MS-based proteomic profiling revealed a significant decrease in spliceosomal proteins inside cancer cells after chemotherapy. Interestingly, we also observed DNA damage-induced hyperphosphorylation of spliceosomal proteins. This post-translational modification is known to inhibit splicing catalysis [45–47]. Therefore, these data demonstrate that four independent mechanisms are activated in response to therapy-induced stress to decrease the number of spliceosomal proteins. These processes act at different levels (i.e., affecting gene expression, pre-mRNA splicing, protein post-translational modification and secretion) and are likely complementary to each other. In other words, they occur simultaneously in response to strong cellular stress and may affect more than two thirds of all spliceosomal proteins within the cell [99] (Fig. 4f).

However, it is important to note that downregulation of spliceosomal proteins does not always lead to decreased splicing efficiency. Alternative splicing is regulated by an intricate network of enhancing and inhibiting splicing factors. Therefore, downregulation of a splicing inhibitor protein may in fact lead to increased splicing efficiency. Moreover, multiple studies have demonstrated that depending on context the same protein may both repress and activate splicing, giving rise to complex regulatory relationships [5]. We believe that the therapy-induced downregulation of spliceosomal proteins may lead to highly specific alterations in pre-mRNA splicing and promote the survival of cancer cells after treatment. In our study, we utilized a small molecule splicing inhibitor that impairs the function of spliceosomal machinery. We speculate that it induces dysregulation of pre-mRNA splicing and therefore does not allow cells to fine tune their splicing in response to genotoxic stress. Failure to adjust splicing after DNA damage may in turn promote cell death. In agreement with this hypothesis, we demonstrated that pladienolide B significantly increases cisplatin-induced apoptosis and impedes DNA reparation. This synergetic effect of pladienolide B with cisplatin may allow the use of lower doses of both compounds in cancer treatment and therefore overcome side effects observed in patients during therapy [24].

Several previously published studies have shown that removal of spliceosomal proteins may activate DNA repair in cancer cells. Most recently, the U2/U5/U6 ribonucleoprotein complex of the spliceosome, which participates in the last stage of splicing, was found to dissociate from chromatin in response to UV radiation [19]. In addition, a lack of spliceosomal proteins inside the cell leads to a high level of histone H2AX phosphorylation, which serves as a signal for subsequent DNA repair [53]. However, the exact mechanism of interplay between spliceosomal proteins and DNA repair remains unknown. It is important to mention that a reduction in splicing efficiency does not indicate a total loss of functional transcripts. Cells contain many protein-coding genes that possess only few or even no introns. Multiple studies have shown that mainly intron-poor genes are activated upon exposure to different stresses [100]. Hence, genes with few introns can be efficiently spliced even in the presence of a small number of functional spliceosomes, whereas the expression of genes with a higher number of introns or with weak splicing sites might be downregulated [101].

## Conclusions

Our analysis revealed a novel stress response mechanism that was observed in 101 cell lines under 12 different conditions. After treatment of cancer cells with various drugs, we detected a reduction in the level of active spliceosomal proteins induced by different pathways, such as intron retention, decreased gene expression, phosphorylation, and extracellular export. These processes act at multiple levels and are likely complementary to each other, i.e., they occur in parallel following stress insults and can collectively affect more than two thirds of all spliceosomal proteins within the cell. The downregulation of spliceosomal components through these processes may promote cancer cells survival following therapy. This stress response mechanism can be inhibited by pladienolide B, which significantly increases the sensitivity of cancer cells to cisplatin, and therefore, pladienolide B is a candidate drug to improve the efficiency of cancer therapy.

## Additional files

Additional file 1: Full information regarding the mRNA microarray gene expression datasets used in this study. The dataset title is used in the text as a dataset identifier. (PDF 171 kb)

Additional file 2: Supplementary materials and methods. (PDF 151 kb)

Additional file 3: *(A)* Description of all alternative splicing events in cancer cell lines after therapy. *(B)* Identification of stop codons in transcripts with retained introns, which were detected in at least half of cancer cell lines before and after chemotherapy. *(C)* Description of all alternative splicing events in PDX tumors after different types of therapy. *(D)* Identification of stop codons in transcripts with retained introns, which were detected in PDX tumors before and after chemotherapy. *(E)* Description of alternative splicing events in spliceosomal genes in PDX tumors after different types of therapy. *(F)* Description of insertions which were detected in 7 cell lines (A375, A549, H3122, N87, PC9, RT112, H358) used in our analysis of alternative splicing changes. (XLSX 716 kb)

Additional file 4: **Figure S1.** PCA clustering of splicing inclusion level differences between treated and untreated PDX tumors. Figure S2: Graph representing the common transcription factors GFI1B *(A)* and TARDBP *(B)* that may induce concerted changes in the expression of pairs of splicing- and mitotic-related genes after a course of chemotherapy. Solid black lines connect a pair of co-expressed genes and red lines connect transcription factors with their target genes. Figure S3: Western blotting analysis of U87MG cells and their concentrated secretomes before and after treatment with 30 μM Cisplatin (CP). Figure S4: Pladienolide B increases the sensitivity of cancer cells to Cisplatin. *(A)* Viability assay of U87MG, Hela and MCF-7 cells that were pretreated with 2 nM Pladienolide B (2 days) following treatment with different concentrations of Cisplatin (4 days). *(B)* FACS analysis of caspase 3/7 and SYTOX staining of SKOV3 cells treated with 0.5 nM Pladienolide B, 10 μM Cisplatin or both drugs together. *(C)* Cell cycle analysis of SKOV3 and HT29 cells treated for 3 days with 0.5 nM and 1 nM Pladienolide B, respectively. *(D)* FACS analysis of phospho ATM staining in Hela, A549 and HT29 cells that were cultivated with 1 nM Pladienolide B (2 days) and subsequently treated with the indicated concentrations of Cisplatin (1 day). (PDF 855 kb)

Additional file 5: Results of the enrichment analysis of genes that were differentially expressed in response to different stress factors. (XLSX 171 kb)

Additional file 6: Description of gene clusters identified by the time clusterization analysis. (XLSX 415 kb)

Additional file 7: Proteins and their peptides identified by a proteome analysis of SKOV3 cells prior to and after Cisplatin treatment. (XLSX 8686 kb)

## Abbreviations

A3SS: Alternative 3′ splice site
A5SS: Alternative 5′ splice site
LC-MS/MS: Liquid chromatography tandem-mass spectrometry
MXE: Mutually exclusive exons
NCBI: National Center for Biotechnology Information
PCA: Principal component analysis
PDX: Patient-derived xenograft
RI: Retained intron
SE: Skipped exon
TF: Transcription factor
