# Supplementary material for: Therapy-induced stress response is associated with downregulation of pre-mRNA splicing in cancer cells
Source: Genome Med. 2018 Jun 27;10:49. doi: 10.1186/s13073-018-0557-y (PMC6020472; doi:10.1186/s13073-018-0557-y)
Supplement: Supplementary file 1 — Full information regarding the mRNA microarray gene expression datasets used in this study. The dataset title is used in the text as a dataset identifier. (PDF 171 kb) [file 13073_2018_557_MOESM1_ESM.pdf]

Additional file 1. Full information regarding the mRNA microarray gene expression datasets used in this study. The dataset title is used in the text as a dataset identifier.

Dataset title	Microarray platform	Cell lines	Time of incubation	Stressor	Number of control samples	Number of treated samples
E-GEOD-66493	Affymetrix GeneChip Human Gene 1.0	U87	24 h.	Cisplatin	3	3
E-GEOD-38122	Affymetrix GeneChip Human Genome U133 Plus 2.0	HepG2	24 h.	Cisplatin	3	3
GSE47980	Illumina HumanRef -8 v3.0	MM200, IgR3, Me4405, Mel-RM, Sk-mel-28	24 h.	Cisplatin	9	9
GSE38545	Illumina HumanRef -8 Expression	SK-OV-3	24 h.	Cisplatin	3	3
GSE24589	Illumina humanRef-8 v1.0 expression	SK-OV-3, OVCAR-3, TOV-21G	24 h.	Cisplatin	8	8
GSE13525	Affymetrix GeneChip Human Genome U133 Plus 2.0	36M2	24 h.	Carboplatin	2	2
E-GEOD-8057	Affymetrix Human Genome U95 Version 2 Array	A2780	24 h.	Oxaliplatin	4	4
GSE51952	Affymetrix GeneChip Human Genome U133 Plus 2.0	HepG2	24 h.	Cisplatin	3	3

GDS3910	Affymetrix GeneChip Human Genome U133 Plus 2.0	MCF-7	11 h.	Cisplatin	2	2
E-MTAB-3645	Affymetrix Human Gene 1.0 ST Array	A2780	72 h.	Cisplatin	3	3
GSE47856	Affymetrix Human Gene 1.0 ST Array	A2008, A2780, A2780cisR, C13, Caov-2, Caov-3, CH1, DOV13, DOV13A, DOV13B, FU-OV-1, Hey, HeyA8, HeyC2, IGROV-1, JHOS-2, JHOS-3, M41, OAW28, OAW42, OV56, OV90, ovary1847, OVCA420, OVCA429, OVCA432, OVCA433, OVCAR-10, OVCAR-2, OVCAR-3, OVCAR-5, OVCAR-8, OVK-18, PA-1, PEO1, RMG-I, RMG-II, SKOV-3, SKOV-4, SKOV-6, SKOV-8, TAYA, TOV-112D, TOV-21G, TYK-nu, UWB1.289	48 h.	Cisplatin	82	89
E-GEOD-50831	Affymetrix GeneChip Human Genome U133 Plus	CaOv3, COV434, EFO-21, OVSAHO, OVCAR-4, OVTOKO,	24 h.	Paclitaxel	63	63

	2.0	KURAMOCHI, OV-90, OVCAR-3, EFO-27, COV644, A2780, COLO 720E, COLO-704, COV504, COV362, OV56, TOV-112D, OVISE, TOV-21G, SK-OV-3				
E-GEOD-50830	Affymetrix GeneChip Human Genome U133 Plus 2.0	SNG-M, HEC-1-B, KLE, RL95-2, MFE-280, HEC-6, HEC-1, AN3 CA, HEC-265, HEC-50B, SNG-II, MFE-296, HEC-59, HEC-151, HEC-251, ECC-1, HEC-1-A, HEC-108, HEC-88nu	24 h.	Paclitaxel	55	57
E-GEOD-50811	Affymetrix GeneChip Human Genome U133 Plus 2.0	UACC-812, HCC-70, HCC-1500, HCC-1419, MDA-MB-453, HCC-1428, MDA-MB-468, UACC-893, AU565, HCC-38, MDA-MB-231, HCC-1143, HCC-1937, DU4475, BT-474, HCC-2218, SKBR3, HCC-1806, HCC-1954, MCF-7, T47D, ZRT, HS578T, BT-549, MDA-MB-436, CAL-51, BT-20	24 h.	Paclitaxel	79	81
E-GEOD-	Affymetrix	G361, HepG2,	6 h.	Irradiation	12	12

30240	GeneChip Human Genome U133 Plus 2.0	TK6, U2OS				
E-GEOD-59732	Affymetrix GeneChip Human Genome U133 Plus 2.0	AU565, BT474, BT549, CAMA1, DKAT, HBL100, HCC1954, MCF10A, MCF12A, MCF7, MDA-MB-231, SKBR3, SUM149, SUM159, T47D, ZR751	24 h.	Irradiation	48	48
E-GEOD-18494	Affymetrix GeneChip Human Genome U133 Plus 2.0	HepG2, U87, MDA-MB231	12 h.	Hypoxia	9	9
E-GEOD-53012	Affymetrix GeneChip Human Genome U133 Plus 2.0	PC-3, SK-OV-3, WM793B	72 h.	Hypoxia	9	9
E-MTAB-3645	Affymetrix GeneChip Human Gene 1.0 ST Array	A2780	72 h.	Hypoxia	3	3
E-GEOD-17188	Agilent Whole Human Genome Microarray 4x44K 014850 G4112F	MDA-MB-231, SCP2, LM2	24 h.	Hypoxia	4	4
E-GEOD-20854	Affymetrix GeneChip Human Genome U133 Plus	Ishikawa H, Hec50co	24 h.	Gefitinib	4	4

	2.0					
E-TABM-585	Affymetrix GeneChip HT Human Genome U133A HT_HG-U133A	A549	20 h.	Dasatinib, Imatinib, Nilotinib	21	27
E-GEOD-47013	Affymetrix Human Exon 1.0 ST Array	MLN120B	-	Doxorubicin	3	3
E-GEOD-13477	Affymetrix Human Genome U133 Plus 2.0 Array	MCF7	24 h.	Doxorubicin	2	2
E-GEOD-19638	Affymetrix Human Genome U133 Plus 2.0 Array	MCF7	-	Doxorubicin	2	2
E-GEOD-39870	Affymetrix Human Genome U133 Plus 2.0 Array	MCF7	-	Doxorubicin	3	3
E-GEOD-59861	GeneChip® PrimeView ™ Human Gene Expression Array	AG 1522 human fibroblasts	3h, 6h, 12h, 24h	Irradiation	3	3,3,3,3
