# Supplementary material for: Therapy-induced stress response is associated with downregulation of pre-mRNA splicing in cancer cells
Source: Genome Med. 2018 Jun 27;10:49. doi: 10.1186/s13073-018-0557-y (PMC6020472; doi:10.1186/s13073-018-0557-y)
Supplement: Supplementary file 2 — Supplementary materials and methods. (PDF 151 kb) [file 13073_2018_557_MOESM2_ESM.pdf]

Additional file 2. Supplementary materials and methods

Pathway Analysis

For functional annotation of differentially expressed genes, we used KEGG [1] and Reactome [2] databases together with the clusterprofiler [3] and ReactomePA [4] R/Bioconductor packages. To analyze the representation of metabolic pathways, redseq_id identifiers were transformed to entrez_id using bioDBnet service [5]. Differentially spliced genes were functionally annotated in accordance with Gene Ontology terms using STRING database [6]. A pvalue correction for multiple testing was made using FDR method, and the cutoff threshold was 0.05.

Time Clusterization Analysis

Time clusterization analysis was performed on three datasets from NCBI GEO with time-course data: E-GEOD-59861, E-GEOD-8057, and E-GEOD-18494 (Table 1, Additional file 1). The fuzzy c-means clustering algorithm by mfuzz R/Bioconductor package [7] divided each dataset based on 15 expression patterns. For the fuzzy c-means clusterization, we selected only probe_ids that significantly changed expression compared with the zero time point at least at one other time point (limma pvalue < 0.05). For each gene_symbol, we left only probe_id with the most markedly changed expression levels between the zero point and the last time point to eliminate degeneracy of probe_id relative to gene_symbol. We were most interested in clusters with genes of the KEGG pathway "Spliceosome" differentially presented (pvalue < 0.05).

Co-regulation Analysis

We performed co-regulation analysis of differentially expressed splicing- and mitotic-related genes from four datasets that described the effects of platinum agents on different cancer cell lines: E-GEOD-66493, GSE13525, GSE66493 and GSE47856 (Table 1, Additional file 1). For each dataset we independently identified differentially expressed splicing and mitotic-related genes and chose pairs between them (consists of one splicing- and one mitotic-related gene) with Spearman's correlation coefficients greater than 0.7 and FDR-corrected pvalue < 0.05. To identify gene pairs that were regulated by common transcription factors (TFs), we created a list of TF associations with the splicing- and mitotic-related genes. To do this, we downloaded data of the merged peaks from database ReMap [8], which consist of overlapping binding sites for identical TFs from public NCBI GEO and ENCODE ChiP-seq datasets. We defined a gene association with a TF if the TF ChiP-seq peak was located within

1 kb upstream and 1 kb downstream of a gene transcription start site. Annotation of ChiP-seq peaks was carried out using the ChIPseeker R/Bioconductor package [9]. To estimate whether the co-expression of a gene pair (splicing- and mitotic-related gene) was significantly associated with their simultaneous regulation by a certain TF, we performed Fisher's exact test. Pvalue and confidence interval odds ratio were calculated for each TF, and a correction for multiple testing was made.

SDS-PAGE

Collected SKOV3 cells before and after Cisplatin treatment were washed by PBS buffer three times. Cell pellets were resuspended in SDS reducing buffer (62.5 mM Tris-HCl (pH=6.8), 20% Glycerol, 2% SDS, 100 mM DTT) and subjected to sonification. Protein concentrations were determined using the Quick Start Bradford protein assay (Bio-Rad) according to the manufacturer's protocol. Cell lysates were separated via 9% (w/v) SDS-PAGE (20 cm × 20 cm) to prefractionate the proteins. Electrophoresis was stopped when the dye front reached 5 cm below the stacking gel. Subsequently, the gel was divided into slices; they was subjected to in-gel protein digestion, and extracted peptide samples were analyzed via LC-MS/MS for protein identification.

In-gel Trypsin Digestion of Cell Lysates For LC-MS/MS Analysis

Gel samples were cut into small (1 mm × 1 mm) pieces and transferred into sample tubes. Protein disulfide bonds were reduced with 10 mM DTT (in 100 mM ammonium bicarbonate buffer) at 50°C for 30 min and afterward alkylated with 55 mM iodoacetamide (in 100 mM ammonium bicarbonate buffer) at room temperature for 20 min in the dark. After alkylation, the gel samples were destained with 50% ACN (in 50 mM ammonium bicarbonate buffer) and dehydrated by the addition of 100% ACN. After removal of the 100% ACN, the samples were subjected to in-gel trypsin digestion. The digestion buffer contained 13 ng/µl trypsin (in 50 mM ammonium bicarbonate buffer). The trypsin digestion proceeded overnight at 37°C. The resulting tryptic peptides were extracted from the gel via the addition of two volumes of 0.5% TFA to the samples (incubation for 1 h) and then two volumes of 50% ACN (incubation for 1 h). Finally, the extracted peptides were dried in vacuum and redissolved in 3% ACN with 0.1% formic acid solution prior to LC-MS/MS analysis.

LC-MS/MS Data Analysis

Raw LC-MS/MS data were converted to .mgf peaklists with the ProteinPilot (version 4.5). For this procedure we run ProteinPilot in identification mode with the following parameters: Cys alkylation by iodoacetamide, trypsin digestion, TripleTOF 5600 instrument, thorough ID

search with detected protein threshold 95.0% against UniProt Protein knowledge base, taxon human (http://www.uniprot.org, with 176397 entries). For thorough protein identification the generated peaklists were searched with MASCOT (version 2.5.1, Matrix Science Ltd) and X! Tandem (CYCLONE, 2013.2.01, The Global Proteome Machine Organization) search engines against UniProt Protein knowledge base, taxon human, with concatenated reverse decoy dataset (with 352794 entries altogether). Precursor and fragment mass tolerance were set at 20 ppm and 0.04 Da, respectively. Database searching parameters included the following: tryptic digestion with 1 possible miss cleavage, static modifications for carbamidomethyl (C) and dynamic/flexible modifications for oxidation (M). For X! Tandem we also selected parameters that allowed a quick check for protein N-terminal residue acetylation, peptide N-terminal glutamine ammonia loss or peptide N-terminal glutamic acid water loss. Result files were submitted to the Scaffold 4 software (version 4.2.1, Proteome Software, Inc) for validation and meta analysis. We used LFDR scoring algorithm with standard experiment wide protein grouping. For the evaluation of peptide and protein hits, a false discovery rate of 5% was selected for both. False positive identifications were based on reverse database analysis.

Cell Proliferation Assay

Cancer cells were plated in 5000 cells per well of 96-well plate, allowed to adhere overnight, and then incubated with either DMSO or 2 nM Pladienolide B for 48 h. Then cisplatin was added to a final concentration of 0, 0.1, 0.3, 1, 3, 10, 30 or 100 μM. After 4 days incubation viability of cells was determined by MTT reagent (Sigma) using Bio-Rad iMark Microplate Reader.

Flow Cytometry

Cells were plated in 6-well plates, allowed to adhere overnight, and then incubated with indicated concentrations Pladienolide B for 48 h. Next, Cisplatin was added and cells were incubated for additional 24 h. Apoptosis was determined using CellEvent Caspase 3/7 Green Cytometry Assay kit (Thermo Fisher) according to the manufacturer's protocol. Cells were analysed on a FACSAria III flow cytometer (BD Biosciences).

For ATM phosphorylation assay, FACS analysis was performed as previously described (Pavlyukov et al. 2011). Briefly, cancer cells were fixed by 4% PFA, permeabilized with 0.2% Triton-X, blocked with serum-free protein block solution (Dako) and incubated with primary anti-phosphoATM (S1981) antibody (ab81292, Abcam) for 1 h following incubation for an additional hour with Alexa Fluor 488 conjugated secondary antibody. After staining,

samples were analyzed by NovoCyte Flow Cytometer (ACEA Biosciences) and the data were processed with FlowJo 10 software.

For cell cycle analysis cells were treated with Pladienolide B for 3 days and then cell cycle distribution were determined as previously described [10]. Briefly, cells were fixed in 70% Ethanol solution for 1 h, and washed with PBS. Then cells were resuspended in 0.5 mL DAPI staining solution and incubated for 30 min on ice in dark. After staining, samples were analyzed by NovoCyte Flow Cytometer (ACEA Biosciences) and the data were processed with FlowJo 10 software.

Immunofluorescence Analysis

Immunocytochemistry (ICC) was performed as described previously (Cheng et al. 2016). Briefly, cells were fixed with 4% PFA, permeabilized with 0.2% Triton-X, blocked with serum-free protein block solution (Dako) and incubated with anti-phosphoATM (S1981) antibody (ab81292, Abcam) for 1 hour at room temperature. Next cells were incubated with Alexa Fluor 488 conjugated secondary antibody for 1 h at room temperature and mounted in DAPI solution. Images were captured with Nikon Diaphot 300 inverted fluorescence microscope (Nikon).

Western Blotting

Cells and their concentrated secretomes were lysed for 30 min on ice in RIPA buffer (Sigma) containing 1% protease inhibitor cocktails (Sigma). Lysates were pre cleaned by centrifugation at 16 000g, 15 min, 4°C. Protein concentration was determined by Bradford assay. Equal amounts of protein lysates (10 µg/lane) were fractionated by 10% SDS-PAGE and transferred to a PVDF membrane (Thermo scientific). Subsequently, the membrane was blocked with 5% Blotting Grade Blocker Nonfat Dry Milk (Bio-Rad) for 1 hour and then incubated with primary antibodies against U2AF1 (ab172614, Abcam), U2AF2 (ab37530, Abcam), RBM11 (ab69358, Abcam) overnight and next incubated with peroxidase conjugated secondary antibodies (GE Healthcare) for 1 hour. Immunolabeled proteins were detected by a chemiluminescence detection system (GE Healthcare).

References

1. Kanehisa M, Goto S, Sato Y, Furumichi M, Tanabe M. KEGG for integration and interpretation of large-scale molecular data sets. Nucleic Acids Res. 2012;40:D109–14.

2. Matthews L, Gopinath G, Gillespie M, Caudy M, Croft D, de Bono B, et al. Reactome knowledgebase of human biological pathways and processes. Nucleic Acids Res. 2009;37:D619–22.

3. Yu G, Wang L-G, Han Y, He Q-Y. clusterProfiler: an R package for comparing biological themes

among gene clusters. OMICS. 2012;16:284–7.

4. Yu G, He Q-Y. ReactomePA: an R/Bioconductor package for reactome pathway analysis and visualization. Mol. Biosyst. 2016;12:477–9.

5. Mudunuri U, Che A, Yi M, Stephens RM. bioDBnet: the biological database network. Bioinformatics. 2009;25:555–6.

6. Szklarczyk D, Franceschini A, Wyder S, Forslund K, Heller D, Huerte-Cepas J, et al. STRING v10: protein–protein interaction networks, integrated over the tree of life. Nucleic Acids Res. [Internet]. 2014; Available from: http://nar.oxfordjournals.org/content/early/2014/10/28/nar.gku1003.abstract

7. Kumar L, E Futschik M. Mfuzz: a software package for soft clustering of microarray data. Bioinformation. 2007;2:5–7.

8. Chèneby J, Gheorghe M, Artufel M, Mathelier A, Ballester B. ReMap 2018: an updated atlas of regulatory regions from an integrative analysis of DNA-binding ChIP-seq experiments. Nucleic Acids Res. 2018;46:D267–75.

9. Yu G, Wang L-G, He Q-Y. ChIPseeker: an R/Bioconductor package for ChIP peak annotation, comparison and visualization. Bioinformatics. 2015;31:2382–3.

10. Darzynkiewicz Z, Juan G, Bedner E. Determining cell cycle stages by flow cytometry. Curr. Protoc. Cell Biol. 2001;Chapter 8:Unit 8.4.
