# Supplementary material for: Therapy-induced stress response is associated with downregulation of pre-mRNA splicing in cancer cells
Source: Genome Med. 2018 Jun 27;10:49. doi: 10.1186/s13073-018-0557-y (PMC6020472; doi:10.1186/s13073-018-0557-y)
Supplement: Supplementary file 4 — Figure S1. PCA clustering of splicing inclusion level differences between treated and untreated PDX tumors. Figure S2: Graph representing the common transcription factors GFI1B (A) and TARDBP (B) that may induce concerted changes in the expression of pairs of splicing- and mitotic-related genes after a course of chemotherapy. Solid black lines connect a pair of co-expressed genes and red lines connect transcription factors with their target genes. Figure S3: Western blotting analysis of U87MG cells and their concentrated secretomes before and after treatment with 30 μM Cisplatin (CP). Figure S4: Pladienolide B increases the sensitivity of cancer cells to Cisplatin. (A) Viability assay of U87MG, Hela and MCF-7 cells that were pretreated with 2 nM Pladienolide B (2 days) following treatment with different concentrations of Cisplatin (4 days). (B) FACS analysis of caspase 3/7 and SYTOX staining of SKOV3 cells treated with 0.5 nM Pladienolide B, 10 μM Cisplatin or both drugs together. (C) Cell cycle analysis of SKOV3 and HT29 cells treated for 3 days with 0.5 nM and 1 nM Pladienolide B, respectively. (D) FACS analysis of phospho ATM staining in Hela, A549 and HT29 cells that were cultivated with 1 nM Pladienolide B (2 days) and subsequently treated with the indicated concentrations of Cisplatin (1 day). (PDF 855 kb) [file 13073_2018_557_MOESM4_ESM.pdf]

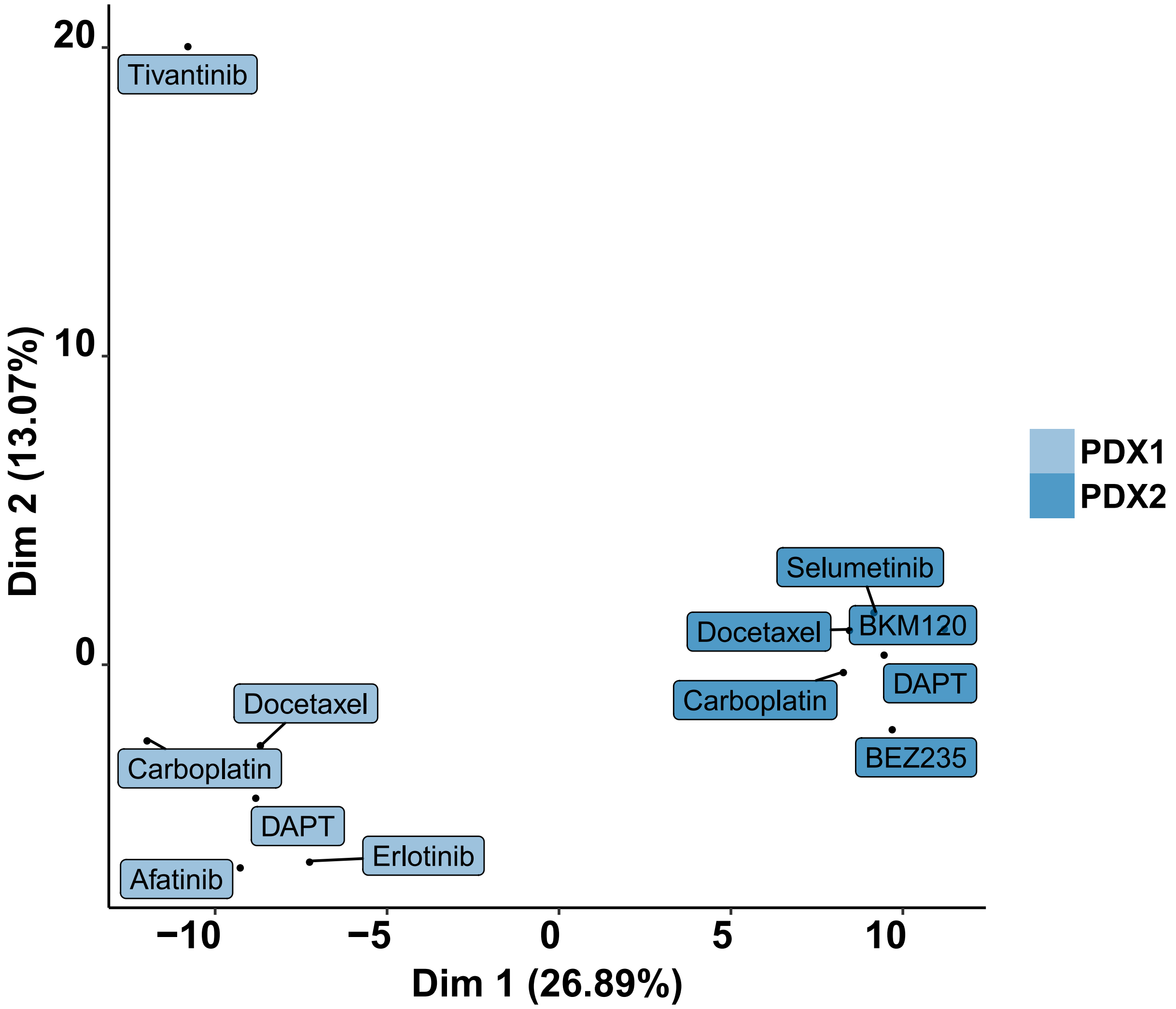

Figure S1: PCA clustering of splicing inclusion level differences between treated and untreated PDX tumors.

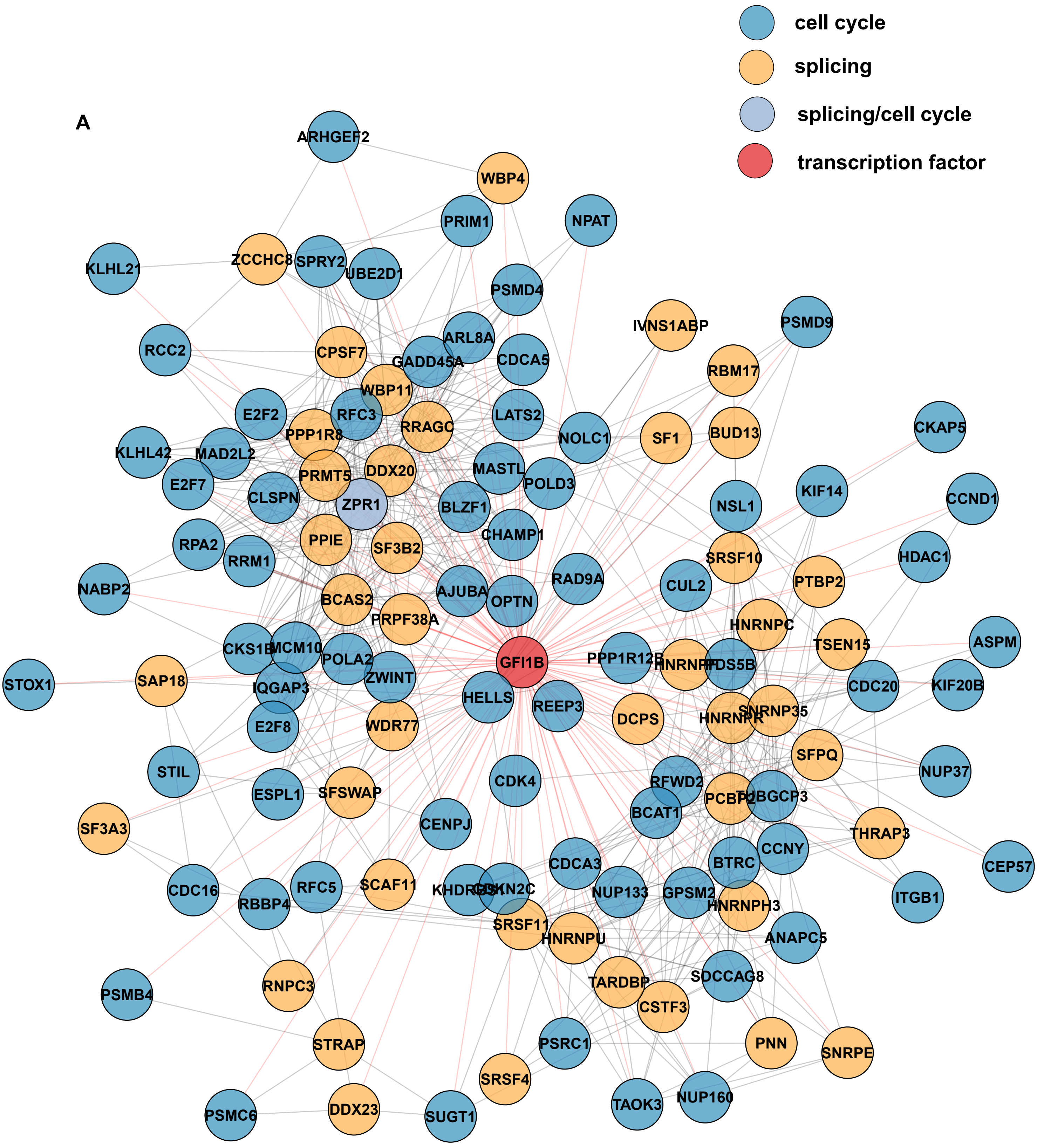

Figure S2: Graph representing common transcription factors GFI1B ***(A)*** and TARDBP ***(B)*** that may induce concerted changes in the expression of pairs of splicing- and mitotic-related genes after a course of chemotherapy. A solid black lines connect a pair of co-expressed genes and red lines connect transcription factors with their target genes.

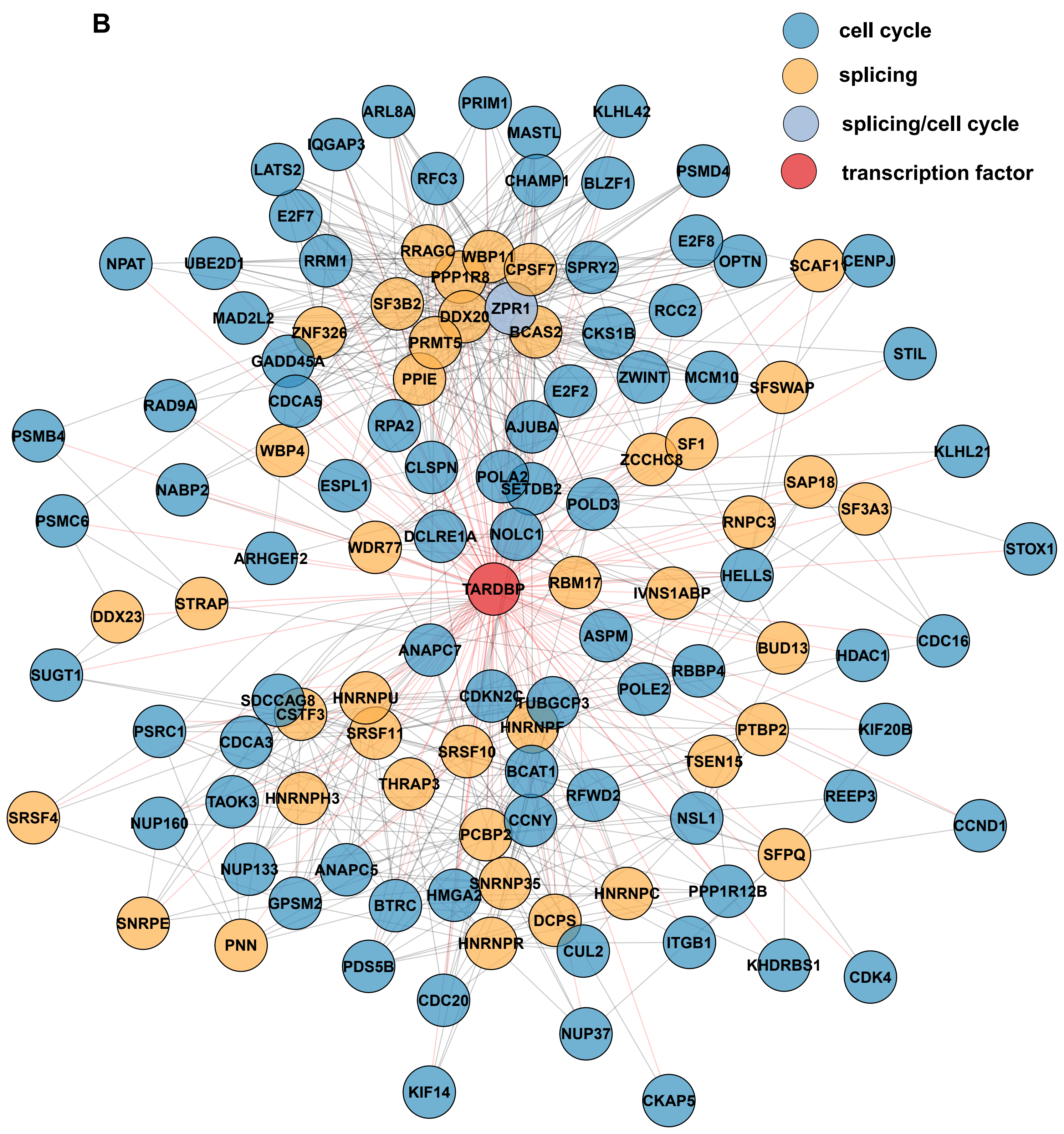

Figure S2: Graph representing common transcription factors GFI1B ***(A)*** and TARDBP ***(B)*** that may induce concerted changes in the expression of pairs of splicing- and mitotic-related genes after a course of chemotherapy. A solid black lines connect a pair of co-expressed genes and red lines connect transcription factors with their target genes.

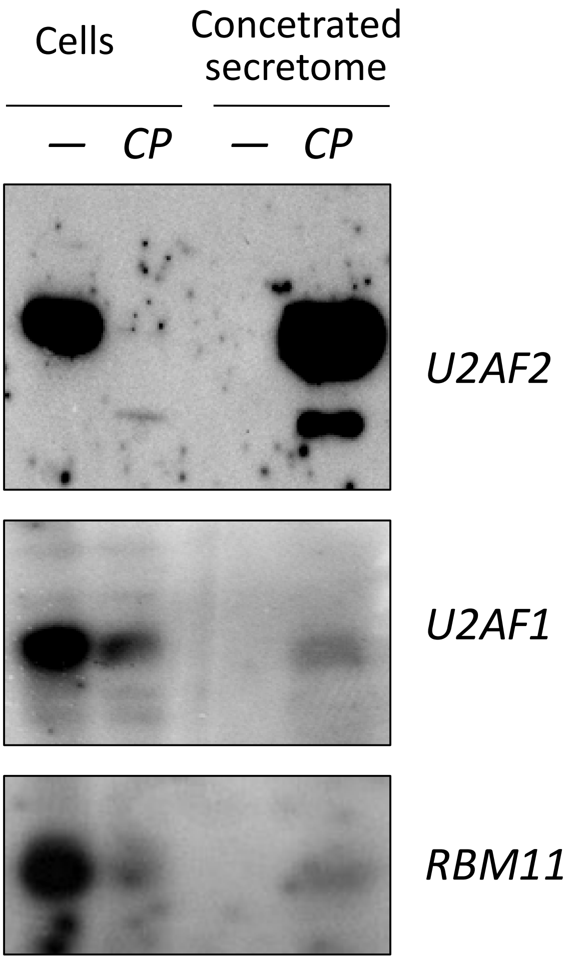

Figure S3: Western blotting analysis of U87MG cells and their concentrated secretomes before and after treatment with 30 μM Cisplatin.

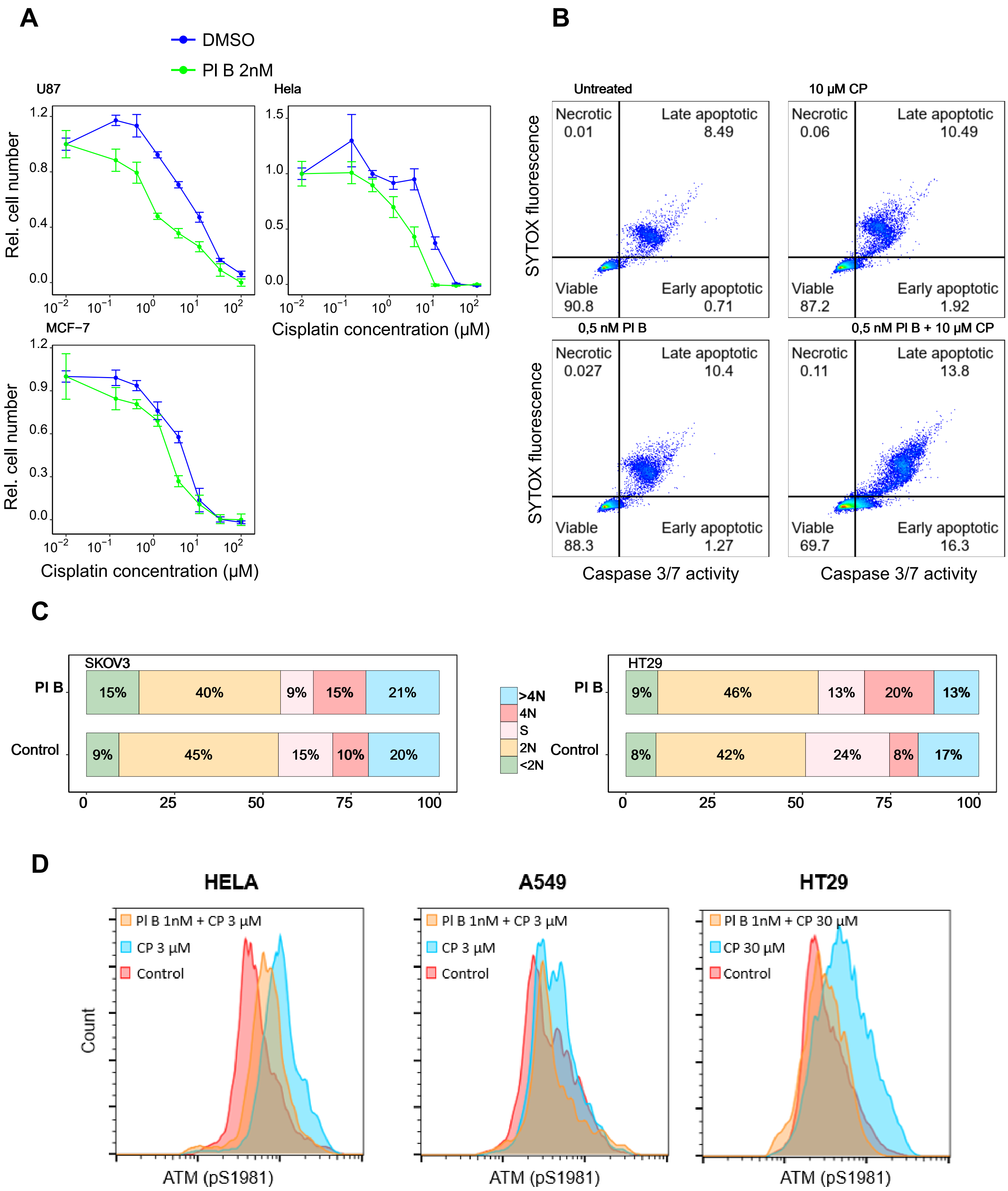

Figure S4: Pladienolide B increases the sensitivity of cancer cells to Cisplatin. ***(A)*** Viability assay of U87MG, Helaand MCF-7 cells that were pretreated with 2 nM of Pladienolide B (2 days) following treatment with different concentrations of Cisplatin (4 days). ***(B)*** FACS analysis of caspase 3/7 and SYTOX staining of SKOV3 cells treated with 0,5 nM Pladienolide B, 10 µMCisplatin or both drugs together. ***(C)***Cell cycle analysis of SKOV3 and HT29 cells treated for 3 days with 0,5 nM and 1 nM of Pladienolide B respectively. ***(D)*** FACS analysis for phospho ATM staining of Hela, A549 and HT29 cells that were cultivated with 1 n M Pladienolide B (2 days) and subsequently treated with indicated concentrations of Cisplatin (1 day).
